# Coat/Tether Interactions—Exception or Rule?

**DOI:** 10.3389/fcell.2016.00044

**Published:** 2016-05-17

**Authors:** Saskia Schröter, Sabrina Beckmann, Hans Dieter Schmitt

**Affiliations:** Neurobiology, Max Planck Institute for Biophysical ChemistryGöttingen, Germany

**Keywords:** vesicle trafficking, coat complexes, protocoatomer, coated vesicles, tethering complex

## Abstract

Coat complexes are important for cargo selection and vesicle formation. Recent evidence suggests that they may also be involved in vesicle targeting. Tethering factors, which form an initial bridge between vesicles and the target membrane, may bind to coat complexes. In this review, we ask whether these coat/tether interactions share some common mechanisms, or whether they are special adaptations to the needs of very specific transport steps. We compare recent findings in two multisubunit tethering complexes, the Dsl1 complex and the HOPS complex, and put them into context with the TRAPP I complex as a prominent example for coat/tether interactions. We explore where coat/tether interactions are found, compare their function and structure, and comment on a possible evolution from a common ancestor of coats and tethers.

## Introduction

In eukaryotic cells, vesicles pass material from one membrane compartment to the other. The cells use elaborate systems of cytoplasmic factors to control the luminal content as well as membrane constituents of the different vesicles (Rothman and Orci, 1992). Factors on the cytosolic side of vesicles and target membrane also impose directionality to the transport process. This is achieved through the recognition of the cargo by cargo receptors at the donor membrane and through the correct identification of the vesicle at the target membrane. Both cargo and vesicle recognition are controlled by small GTPases. GTPases of the ARF family control the cargo selection by coat complexes, while GTPases of the Ypt/Rab family are required for the fusion of vesicles with the appropriate target membrane (Behnia and Munro, 2005).

The main players during vesicle formation are coat complexes. They consist of soluble proteins and are recruited from the cytosol by cargo molecules. The coat formation leads to the bending of the donor membrane and, finally, vesicle release. Members of the SNARE family of membrane proteins are among these cargo molecules (Kuehn et al., 1998; Rein et al., 2002; Lee et al., 2005). They are the actual catalysts of membrane fusion (Söllner et al., 1993). Thus, SNARE proteins have two roles, one in vesicle formation and one in vesicle fusion. The packaging of specific SNAREs into vesicles is one important means to convey vesicle identity and ensure the fusion at the correct target membrane: Each transport step in the cell uses a specific set of SNAREs (McNew et al., 2000). The formation of helical bundles from SNAREs on vesicle and target membrane provides the energy to fuse the apposed membranes. However, SNAREs are not sufficient to guarantee that vesicles find their right target membrane (Brandhorst et al., 2006). Instead, additional components are recruited, often by the combined action of SNAREs and GTPases. These so-called tethering factors are thought to mediate the first contact between membranes that are bound to fuse (Cao et al., 1998; Whyte and Munro, 2002). They bind to proteins on opposite membranes, and at least some of these tethering factors also can bind specific phospholipids directly. Whether the tethering factors in fact form bridges between two different membranes and thus represent real tethers has not been shown for all putative tethering factors (Brunet and Sacher, 2014).

All SNAREs share a strongly conserved core structure, and the tetrameric SNARE complexes assemble mostly in a 3:1 ratio from three SNAREs on the target membrane and one SNARE on the vesicle membrane. Compared to SNAREs, the tethering factors are a much more heterogeneous group of proteins. In particular, the number of subunits making up multisubunit complexes ranges from 3 to 10 and cannot be identified by conserved sequence motifs or easily recognized by Hidden Markov model profiles, as is the case for SNARE proteins (Kloepper et al., 2007). Some Golgi-associated tethering factors are homodimers made up of long coiled–coil proteins (Yu and Hughson, 2010).

Tethering factors (or complexes) interact with SNAREs and/or GTPases to control the specificity of vesicle fusion. In recent years, however, a number of papers came out where evidence was presented that tethering complexes can also interact with coat complexes (Trahey and Hay, 2010; Angers and Merz, 2011). This indicates that cells may use the coats not only for vesicle budding, but they may also keep them as stick-on labels that carry address information.

**Figure 1:**
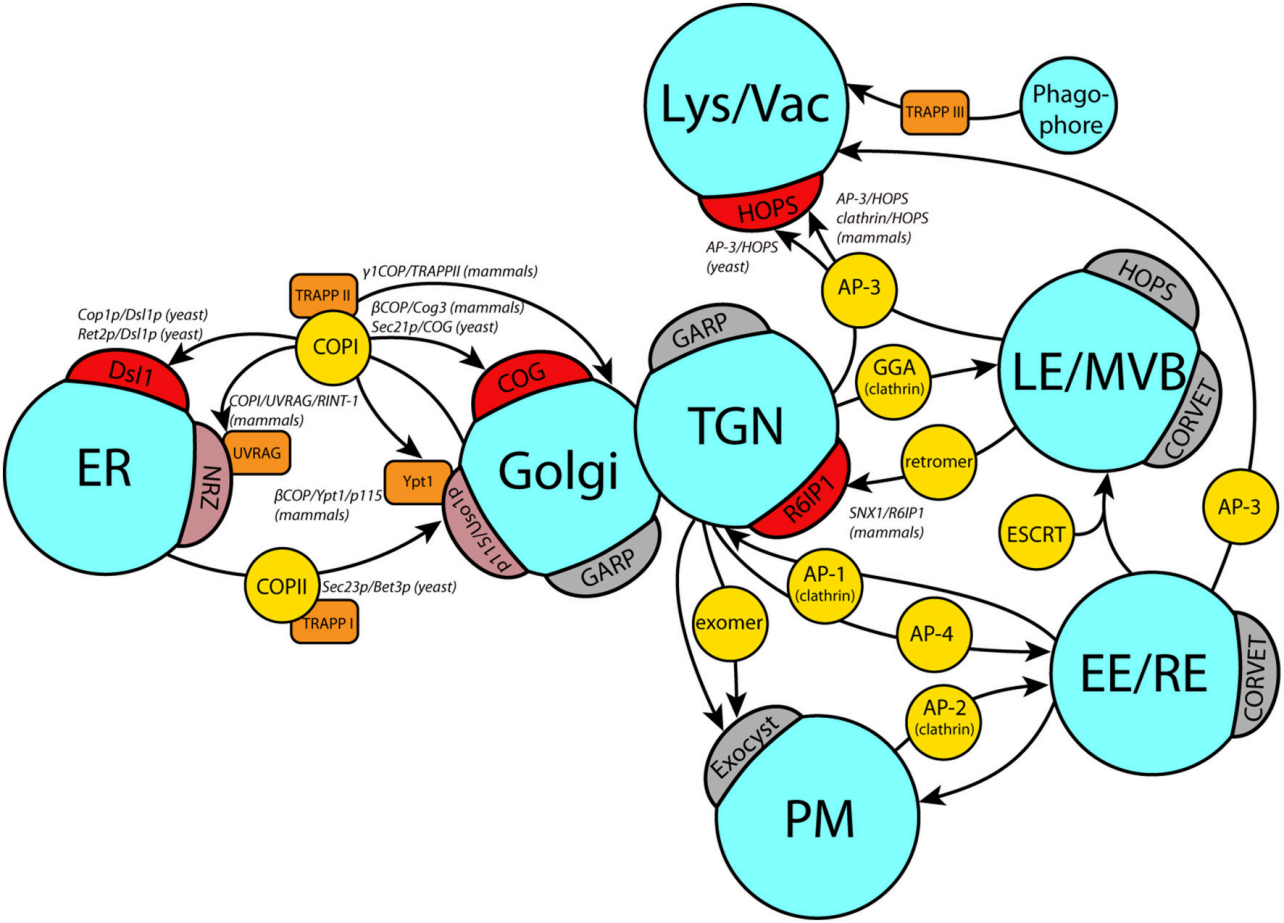
**Coat/tether interactions at different target organelles**. Schematic overview of currently known coat/tether interactions on their respective organelles. Arrows indicate the transport pathway with the involved coats in yellow circles. Direct interactions of coats with tethers on the target membranes (crescents) are marked in red, or light red if they require additional factors (orange boxes). Where known, the precise subunits involved in the coat/tether interaction are listed in italic writing in the order coat subunit/tether subunit. A few selected tethers with no known coat interactions on the respective membrane are depicted in gray crescents. ER, endoplasmic reticulum; TGN, *trans* Golgi network; Lys/Vac, lysosome/vacuole; LE/MVB, late endosome/multivesicular body; EE, early endosome; RE, recycling endosome; PM, plasma membrane.

Beside the coat/tether interactions discussed below in more detail, evidence was presented for a number of other coat/tether interactions. Most of them involve interactions of the COPI coat and different Golgi tethering factors: In yeast, the Trs120p subunit of Golgi tether TRAPP II co-purifies with the COPI subunit α-COP, while experiments with the homologous subunit of the mammalian TRAPP II complex showed interaction with γ-COP but not ε-COP (Yamasaki et al., 2009). γ-COP in yeast (Sec21p) is also the interacting partner of the intra-Golgi COG tethering complex (Suvorova et al., 2002). Analogously, β-COP co-purifies with Cog3 from mammalian cells (Zolov and Lupashin, 2005). A non-COPI/tether interaction was observed in mammalian cells, where the TGN-localized tether Rab6IP1 (Rab6 interacting protein 1) interacts with the retromer component SNX1 (sorting nexin 1; Wassmer et al., 2009). An overview of currently known coat/tether interactions is given in Figure 1.

This review explores whether coat/tether interactions were a fundamental feature of primordial tethering complexes in early eukaryotic cells, or whether they developed independently. We will focus on three prominent coat/tether interactions for our analyses: The interaction of (i) the COPI coat and the Dsl1 complex involved in Golgi to ER retrograde transport, (ii) the AP-3 coat and the HOPS complex, an interaction that mediates direct transport of transmembrane proteins of the limiting vacuolar membrane from the *trans*-Golgi to the lysosome. We will put our findings into context with the interaction of (iii) the COPII coat and the TRAPP I complex, which is required for ER to Golgi forward transport (recent evidence in fact calls into question the classification of TRAPP complexes as tethers, as discussed later). Table 1 lists the constituents of the coats and tethers mentioned, and some of their properties and interaction partners.

**Table 1:** **Composition and properties of coats and tethers involved in transport between ER and Golgi and from the late Golgi to the lysosome in yeast**.

	**COPI / Dsl1**	**COPII / TRAPP I**	**AP-3 / HOPS**
Acronym stands for:	COPI (**co**at **p**rotein complex I) Dsl1 (**d**ependent on **Sl**y1-20)	COPII (**c**oat **p**rotein complex II) TRAPP (**tra**nsport **p**rotein **p**article)	AP-3 (**a**daptor **p**rotein complex 3) HOPS (**ho**motypic fusion and vacuole **p**rotein **s**orting
Transport step	Golgi-ER	ER-Golgi	Golgi-lysosome
Coat proteins	α-, β-, β′-, γ-, δ-, ε-, ζ-COP	Sec23p, Sec24p, Sec13p, Sec31p	Apl5p, Apl6p, Apm3p, Aps3p
Subunits of the tethering complex	Dsl1p, Dsl3(Sec39)p, Tip20p	Bet3p, Bet5p, Trs20p, Trs23p, Trs31p, Trs33p	Vps39p, Vps41p, Vps11p, Vps16p, Vps18p, Vps33p
SNAREs	R-SNARE Sec22p Qa-SNARE Ufe1p Qb-SNARE Sec20p Qc-SNARE Use1p	R-SNARE Sec22p Qa-SNARE Sed5p Qb-SNARE Bos1p Qc-SNARE Bet1p	R-SNARE Ykt6p Qa-SNARE Vam3p Qb-SNARE Vti1p Qc-SNARE Vam7p
Type of MTC	Member of the CATCHR family (**c**omplexes **a**ssociated with **t**ethering **c**ontaining **h**elical **r**ods) including the COG, GARP and exocyst complexes	TRAPP I shares five subunits with the TRAPP II and III complexes which are required for intra-Golgi transport and autophagosome biosynthesis	The HOPS complex is a Class C Vps complex. It shares four subunits (Vps11-33) with the endosomal CORVET complex: Unique CORVAT subunits are Vps3p and Vps8p
Main structural elements	α-helical bundles or “CATCHR domains” (Dsl1p, Tip20p), 18 nm long α-solenoid Dsl3(Sec39)p	Small globular subunits, three of them are longin domains	Five subunits consist of β-propellers followed by an α-solenoid, Vps33p is an SM protein
Conformational changes	Can switch between an Y-shaped and an closed conformation	No change in size and shape	Size can vary between 28 to 40 nm
Size	20 nm long rod in its closed conformation 250 kDa	18 × 6.5 × 5 nm 170 kDa	650 kDa
Biochemical activities		Bet3p, Bet5p, Trs23p, plus Trs31p act as GEF for Ypt1p/Rab1	Yps41p is an effector of the Ypt/Rab GTPase Ypt7p
Kinases involved		Hrr25p	Yck3p
Recruitment to	ER	COPII vesicle	Lysosome or multivesicular body

## Timing and factors of vesicle coat removal

The uncoating of vesicles is initiated during or shortly after scission of the newly formed vesicles, either by the recruitment of uncoating factors or by inactivating GTPases that control the coat formation. Clearly, the presence of a complete coat would prevent the SNARE-mediated fusion of the vesicles with the target membrane. It is thus crucial to examine what is known about the uncoating processes in transport steps, and how this may interplay in a timely manner with a possible recognition of the vesicle through its coat.

The removal of coat elements is best studied for **clathrin**, where the disassembly of the triskelia is catalyzed by the ATPase Hsp70. The uncoating enzyme is recruited to the vesicle by a co-chaperone with binding sites for clathrin and Hsp70, either neuronal auxilin 1 or the ubiquitously expressed GAK/auxilin 2 (Ungewickell et al., 1995; Greener et al., 2000). In clathrin-coated endocytic AP-2 vesicles, where adaptor proteins (AP-2) and cargo can be analyzed by TIRF (total internal reflection fluorescence) microscopy, the removal of the clathrin cage and the adaptor layer may happen independently (Rappoport et al., 2006). A large part of the clathrin disappears from vesicles shortly after scission, before the vesicles move away from the plasma membrane (Massol et al., 2006; Mattheyses et al., 2011). Auxilin is recruited in a burst, shortly before the coat formation is complete (Massol et al., 2006). This observation could point toward a simple stochastically driven loss of the coat. An alternative interpretation, however, is that the observed heterogeneity of the kinetics is due to different populations of clathrin-coated vesicles. Differences in the persistence of the coat may reflect differences in cargo and in the sites to which the vesicles are headed.

AP-1 and AP-3 dependent intracellular transport is more difficult to analyze. Three-dimensional time-lapse movies had to be recorded for AP-1 and AP-3 vesicles, and their lifetime is comparable to that of AP-2 vesicles (Kural et al., 2012). The apparently heterogeneous disassembly behavior of the clathrin cage makes it rather unlikely that clathrin is typically involved in vesicle recognition through tethers at the target membrane. Currently, no evidence exists that the clathrin cage is recognized by tethers or used for recognition at the target membrane, even though this is not a conclusive argument to rule out such a function. The adaptor complexes on the other hand are candidates for coat/tether interactions; and indeed one example of such an interaction has been reported, as discussed later.

Initial evidence for an early removal of the **COP coats** came from the analysis of factors that stimulate the activity of small GTPases required for coat and vesicle formation. The stimulation of their low intrinsic GTPase activity inactivates them and induces their release from the membrane (Pucadyil and Schmid, 2009). In the case of the COPII coat, this activation is achieved by the fully assembled coat itself, where components of the inner and the outer layer of the coat act synergistically on the small GTPase Sar1p (Antonny et al., 1997). In contrast, the GTPase Arf1p that is required for COPI coat assembly is activated by proteins that can sense the curvature of the vesicle membrane (Antonny et al., 2001). Both findings, however, were interpreted in a way that COPI and COPII vesicles lose their coat quite early after fission. However, more recent findings suggest that these results may not fully reflect the physiologically occurring processes. Mutations in subunits of the Dsl1 complex or the HOPS complex as well as mutations in the corresponding SNAREs led to the accumulation of large amounts of coated vesicles (Angers and Merz, 2009; Zink et al., 2009), suggesting that the tethering complexes are involved in uncoating. In addition, CHO cells depleted of a COG subunit accumulate COPI-coated vesicles (Zolov and Lupashin, 2005). These results indicate that vesicles still carry their coat when they arrive at the target membrane, and that coat/tether interactions are possible *in vivo*. The recently determined structure of the COPI coat revealed that the building blocks of the coat, the triads, are connected by those domains that are the binding sites for Dsl1p of the Dsl1 tethering complex. The μ-domain of δ-COP at some linkages, or ε-COP and the C-terminus of α-COP at others (Andag and Schmitt, 2003; Zink et al., 2009; Hsia and Hoelz, 2010; Dodonova et al., 2015; Suckling et al., 2015). This indicates that the Dsl1 complex may be involved in the removal of triads from the vesicle, possibly by competitively binding COPI complexes at these connection sites thus disassembling the coat units. Further proof for this is required since uncoating could not be reconstituted *in vitro* so far. Interestingly though, these findings suggest that the timed coat removal of COPI vesicles differs from that of clathrin coats, at least in yeast: The coat of COPI vesicles appears to be released only after vesicle recognition on the target membrane.

Comparable COPII clusters were only observed in COPII budding mutants, and not in mutants with fusion defects (Shindiapina and Barlowe, 2010). More precisely, the mutation of the COPI tether Uso1p did not lead to accumulation of coated COPII vesicles. However, independent evidence indicates that COPII vesicles may remain partially coated: COPII vesicles that are formed by permeabilized NRK cells carry more than 55% of the inner COPII shell and 15% of the outer COPII shell (Cai et al., 2007; Bentley et al., 2010). Vesicles produced *in vitro* by permeabilized yeast cells also retain most of their coat, as determined for subunits from the inner and outer shell (Lord et al., 2011). The results support the hypothesis that coats or partially present coats on vesicles may be used for identification at the target membrane (Trahey and Hay, 2010).

## Tethering complexes coat the target membrane

The next question concerns the localization of the tethering complexes. If the tethering indeed involves their interaction with the vesicle coat, then the tethering complexes should either be localized at the target membrane, or they must be recruited to it during the tethering process.

**Figure 2:**
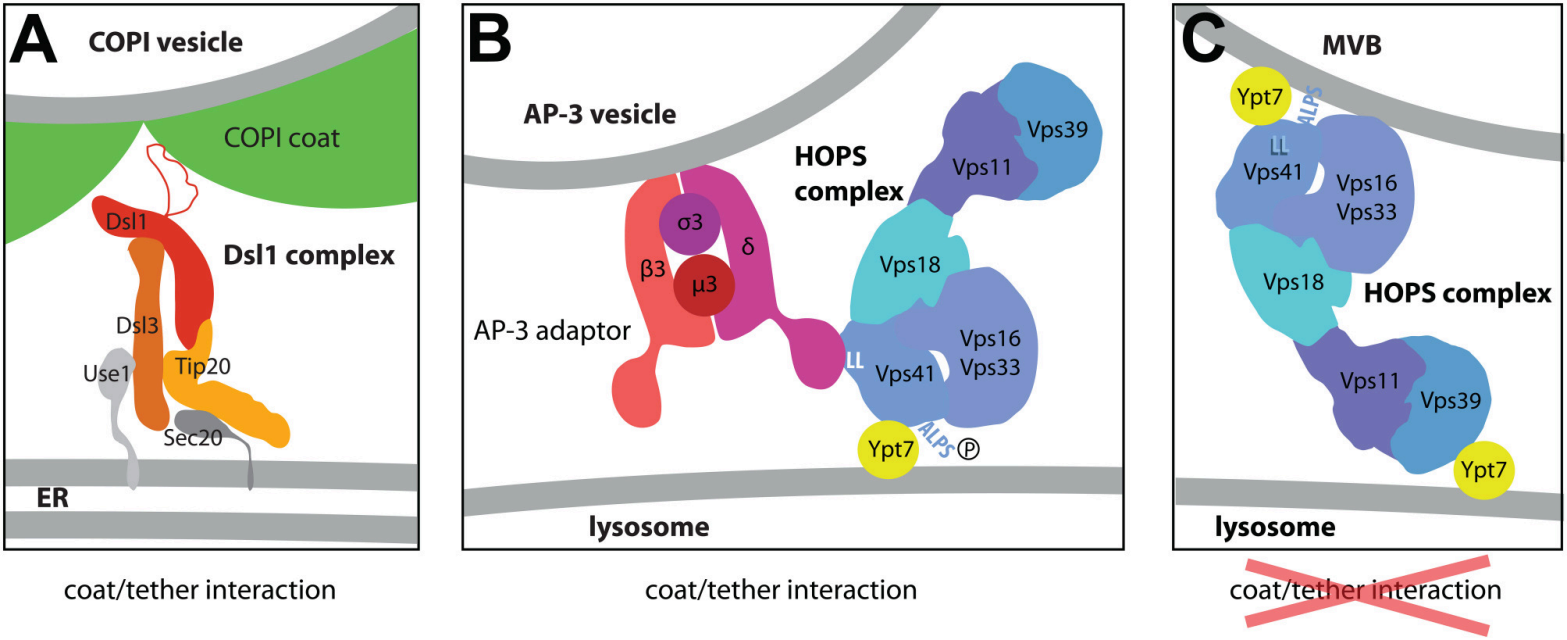
**Models for the interaction of the Dsl1 and HOPS complexes with COPI or AP-3 coats. (A)** The structure of the Dsl tethering according to the structures determined by the Hughson lab (Ren et al., 2009; Tripathi et al., 2009). Dsl1p binds COPI coat via an unstructured lasso domain (Andag and Schmitt, 2003; Ren et al., 2009; Schmitt, 2010; Suckling et al., 2015) at the sites where the coat triads connect (Dodonova et al., 2015). They may represent the sites where coat depolymerization begins. **(B,C)** Two different tethering modes of the HOPS complex at the surface of the lysosome (Cabrera et al., 2010). The arrangement of subunits as shown here was determined by negative stain EM of different HOPS constructs (Kuhlee et al., 2015). **(C)** Depicts the HOPS complex as a tether bridging two membranes very much like in homotypic lysosome-lysosome fusion. At the surface of the multivesicular body, however, the Vps41 subunit can bind to the curved membrane via its curvature-sensing ALPS domain. In this conformation, the binding site for the δ-subunit of the AP-3 complex is masked **(C)**. At the surface of the flat lysosome, in contrast, the ALPS domain cannot bind efficiently to the membrane, (i) due to lower curvature of the membrane and, (ii), since the ALPS motif is phosphorylated by the Yck3 kinase **(B)**. For the interaction with the membrane, HOPS has to rely solely on the interaction of Vps41p with GTP-bound Ypt7p and on the ability of other HOPS subunits to bind to acidic lipids (not illustrated here; Behrmann et al., 2014; Orr et al., 2015). Importantly, in this configuration the δ-subunit of the AP-3 complex has access to its binding site at the Vps41p subunit, a double leucine motif marked as LL in this Figure. By using this specific binding mode, Golgi-derived AP-3 coated vesicles preferentially bind to HOPS complexes at the surface of the lysosome thereby avoiding fusion with endosomes.

The **Dsl1 complex** and its mammalian counterpart, the syntaxin 18 or NRZ complex (Aoki et al., 2009; Civril et al., 2010), are in a very tight complex with the ER-localized SNAREs, and visualization of their constituents gave a pattern consistent with an ER localization (Figure 2A; Reilly et al., 2001; Hirose et al., 2004; Arasaki et al., 2006; Aoki et al., 2009; Meiringer et al., 2011). For additional details on the more complex functional aspects of the mammalian ZW10 complex, see Schmitt (2010).

Similarly, the **HOPS** complex localizes to the target membrane of AP-3 vesicles, the lysosome, but also to late endosomes or multivesicular bodies (MVB) (Nakamura et al., 1997; Cabrera et al., 2010). This reflects the fact that the HOPS is involved in different transport steps: (i) homotypic lysosome/lysosome fusion, (ii) late endosome (MVB)/lysosome fusion and (iii) the fusion of AP-3 coated vesicles with the lysosome (Bowers and Stevens, 2005). This begs the question how the specific localization of tethers is determined.

## Recruitment factors of tethers (1): SNAREs

None of the tethers discussed here has subunits that carry a transmembrane domain. In fact, only a few homodimeric coiled-coil Golgi tethers like giantin are integral membrane proteins (Gillingham and Munro, 2003). Therefore, the sites that the Dsl1, HOPS, and TRAPP complexes are recruited to are determined by additional factors on the surface of the membranes. Here, a very interesting interaction comes into play: Both the Dsl1 and the HOPS complex were shown to bind single SNAREs via N-terminal domains that lie in front of the SNARE domains.

In case of the **Dsl1 complex**, these are the N-terminal domains of the Q_b_-SNARE Sec20p and the Q_c_-SNARE Use1p (Ren et al., 2009; Diefenbacher et al., 2011; Meiringer et al., 2011). Both domains do not show any sequence similarities to other N-terminal domains of SNARE proteins and were not predicted to contain either a lipid binding domain, a longin or H_abc_ α-helical domain. Surprisingly, the formation of only one of these two connections is still sufficient for cell viability (Kraynack et al., 2005; Tripathi et al., 2009). Ren et al. (2009) also observed binding to the fully assembled SNARE complex, while Meiringer et al. (2011) observed very inefficient binding of the full set of SNAREs to the Dsl1 complex *in vitro*. Instead, they found that the lipid-anchored R-SNARE Ykt6p may act as an acceptor for Sec22p, the R-SNARE that associates with the Q-SNAREs at the ER.

The Sly1 proteins may act as additional potential recruiting factors for the Dsl1/NRZ complexes. They are members of the Sec1/Munc18-like protein family (SM), which cooperate with SNAREs during vesicle fusion. The Sly1 proteins act in transport between ER and Golgi, and there is evidence that Sly1p is required for retrograde transport (Reilly et al., 2001; VanRheenen et al., 2001; Li et al., 2005). It associates with the tethering complex in yeast and mammalian cells to varying degrees. It may do so indirectly through its interaction with the SNAREs (Hirose et al., 2004; Kraynack et al., 2005; Li et al., 2005).

The **HOPS complex** also binds to individual SNARE proteins, and contains a stably associated SNARE interacting SM protein (Seals et al., 2000). It binds the Q_a_-SNARE Vam3p via its N-terminal H_abc_-domain, and the Q_c_-SNARE Vam7p via the PX lipid-binding domain (Krämer and Ungermann, 2011; Lobingier and Merz, 2012; Lürick et al., 2015). In addition, the HOPS complex can interact with partially or fully assembled bundles of the SNARE domains (Baker et al., 2015; Lürick et al., 2015). This interaction is mediated by the stable HOPS subunit Vps33p, a member of the Sec1/Munc18 protein (SM) family. Vps33p, unlike other SM proteins like Vps45 and Munc18, is not able to recognize the N-terminal peptide or the H_abc_ domain of Vam3p on its own, and requires Vps16p in addition to fulfill this task (Lürick et al., 2015). The same subunit plus Vps18p is involved in binding of the PX-domain of Vam7p (Krämer and Ungermann, 2011).

In summary, both the Dsl1 and the HOPS complexes are able to bind to individual SNAREs as well as assembled SNARE complexes (Ren et al., 2009; Krämer and Ungermann, 2011; Lobingier and Merz, 2012; Baker et al., 2015). This suggests that their function lasts across the SNARE zippering process, possibly by acting as SNARE complex assembly factors (Baker et al., 2015).

As mentioned above, SNAREs are promiscuous, so additional recruiting factors are needed. In fact, the tethering complex/SNARE interactions may not be essential for the actual tethering step (Hickey and Wickner, 2010), since the HOPS complex can rely on GTPase or direct binding to lipids for the docking of membranes, as discussed in the next paragraph.

## Recruitment factors of tethers (2): small GTPases and lipids

Another class of proteins that are both key to membrane identity and act as recruiting factors for tethering complexes are the small GTPases of the Ypt/Rab family. For the **Dsl1 complex**-dependent retrograde transport, the Spang lab has used a *YPT1* (Rab1) knock-out strain to show that this GTPase is not only required for forward transport, but is also involved in Golgi-ER retrograde transport in yeast (Kamena et al., 2008). Mammalian cells express a much larger set of Ypt/Rab GTPases, and the retrograde transport is regulated by a specialized small GTPase Rab18. Evidence for an involvement of Rab/Ypt GTPase in the tethering process of COPI vesicles in mammalian cells was presented recently by Gillingham et al. (2014) who identified the Dsl1/NRZ complex as an effector of Rab18.

For the **HOPS complex**-dependent tethering to vacuolar membranes, the small Ypt/Rab GTPase Ypt7p was shown to be more important than the interaction of HOPS with SNAREs (Hickey et al., 2009; Hickey and Wickner, 2010). In fact, the HOPS complex is an effector of Ypt7p, since it preferentially binds to the GTP-bound form of this small GTPase (Price et al., 2000) In addition, the HOPS complex can bind to the membrane directly via the head groups of phosphoinositides and other acidic lipids (Stroupe et al., 2006; Behrmann et al., 2014; Orr et al., 2015).

The HOPS complex seems to be ideally suited as a tether since it carries two different Ypt7-binding subunits, Vps39p and Vps41p (also known as Vam6p and Vam2p; Price et al., 2000; Brett et al., 2008). Both are positioned at the opposing ends of its elongated structure (Bröcker et al., 2012). Thus, the HOPS complex can form a bridge between late endosomes or multivesicular bodies (MVBs) on one side and the lysosome on the other side by interacting with Ypt7 GTPases on the opposing membranes (Ho and Stroupe, 2015). Besides this two-armed tethering mode, the lysosome-localized HOPS complex can also act as a receptor for AP-3 vesicles via Apl5p, the δ-adaptin-like subunit of the AP-3 coat (Rehling et al., 1999; Angers and Merz, 2009; Figure 2B). This tethering mode requires the presence of Ypt7p only on the target membrane. Transport via the AP-3-coated vesicles is involved in a special transport route between Golgi and lysosome, by which yeast cells avoid fusion of vesicles carrying lysosome-bound membrane proteins with multivesicular bodies. In yeast, this is achieved by direct vesicular transport from the Golgi to the lysosome via AP-3 vesicles. A typical cargo for this step is the enzyme alkaline phosphatase (ALP, Pho8p). Some other cargo proteins are themselves part of the targeting machinery, like the SNAREs Vam3p and Nyv1p and the lipid-anchored Type I casein kinase Yck3p (Cowles et al., 1997; Ostrowicz et al., 2008). The AP-3 transport route prevents these proteins from being internalized by the MVBs or from becoming active at the surface of endosomes.

How can the HOPS complex switch between the different tethering modes? One obvious possibility is through posttranslational modifications. The phosphorylation of the HOPS complex changes the dependence of HOPS on Ypt7-GTP for its tethering function (Brett et al., 2008; Cabrera et al., 2009; Ho and Stroupe, 2015). Recent *in vitro* experiments with liposomes clearly showed that the phosphorylated HOPS requires Ypt7-GTP for tethering, while non-phosphorylated HOPS complex is active if Ypt7-GDP is present on both membranes (Zick and Wickner, 2012; Ho and Stroupe, 2015). This antagonistic relationship between phosphorylation and the GTP/GDP status suggests that the phosphorylation adjusts the balance between the different membrane recruitment modes for HOPS.

Significantly, the enzyme that phosphorylates the HOPS complex was mentioned above already as cargo of the AP-3 pathway. This membrane-anchored kinase, Yck3p, depends on this transport route for its proper localization to the lysosome (Sun et al., 2004; LaGrassa and Ungermann, 2005). At the same time, Yck3p was shown to be as equally required for proper functioning of the AP-3 pathway as the subunits of the AP-3 coat (Anand et al., 2009; Cabrera et al., 2009). A clue as to how Yck3p can mechanistically bring about the switch in tethering modes of the HOPS complex came when Christian Ungermann's lab identified the phosphorylation sites and the AP-3 binding site within HOPS. Both are located in the N-terminal part of the Ypt7p-interacting subunit Vps41p (Cabrera et al., 2009, 2010). The binding site for the AP-3 subunit Apl5p lies in front of the putative β-propeller domain (Cabrera et al., 2010), while the phosphosites lie within an α-helical region that is adjacent to the opposite end the putative β-propeller (Cabrera et al., 2009). This means that the phosphosites and the Apl5p-binding site come close to each other in the Vps41 protein and thus may affect each other directly (The same region is also involved in the binding of Ypt7p).

The α-helical region was predicted to constitute a so-called ArfGAP1 lipid packing sensor or amphipathic lipid packing sensor (ALPS) motif (Drin et al., 2007). Phosphomimetic mutations in this domain prevent membrane binding *in vivo* and *in vitro* (Cabrera et al., 2009, 2010). The binding to small liposomes is also prevented when Vps41p is pre-incubated with Apl5p (Cabrera et al., 2010). Thus, the N-terminus of Vps41p can adopt two conformations, one in which Vps41p binds to the membrane directly (without the help of Ypt7p) and does not allow binding of the AP-3 coat. In the second conformation, phosphorylation of the ALPS motif prevents binding to the membrane and exposes the AP-3 binding site. Two factors favor the transition on the first conformation to the second: the low curvature of the lysosomal membrane and the presence of the Yck3p on the surface of the lysosome. According to the model proposed by Cabrera et al. (2010), transition between these two states occurs after the recruitment of endosomes/MVBs to the lysosome. Since the endosomes/MVBs have a diameter of 100 nm (Luhtala and Odorizzi, 2004; Balderhaar and Ungermann, 2013), the membrane curvature is low and Yps41p can bind directly to it, while the AP-3 binding site is masked (Figure 2C). After fusion with the lysosome, Vps41p loses its contact with the flat lysosomal membrane, the ALPS motif can be phosphorylated, thereby lowering the affinity of the ALPS domain for membranes even more. As a consequence, the binding site for the AP-3 complex becomes accessible. This ensures that the coat/tether interaction occurs only at the surface of the lysosome. Thus, the yeast cells use a sophisticated mechanism to prevent AP-3 vesicles from fusing with MVBs. They turn a two-armed tether, which links the membranes of MVB and lysosome, to a monovalent tether that uses a coat complex as additional linker to the second membrane.

## Conservation of coat/tether interactions between yeast and mammalian cells

The interaction between COPI and Dsl1p was first described in 2001 in *Saccharomyces cerevisiae* (Andag et al., 2001; Reilly et al., 2001). An unstructured domain within the largest subunit of the Dsl1 complex, Dsl1p itself, is required for the binding to the COPI coat (Andag and Schmitt, 2003; Ren et al., 2009). This so-called lasso domain is exposed at the tip of the whole complex approximately 20 nm above the membrane surface (Ren et al., 2009) and binds to δ-COP and α-COP via tryptophan-containing binding motifs (Andag and Schmitt, 2003). The structure of a δ-COP fragment in complex with such a W × W motif was recently determined by Suckling et al. (2015). Another subunit of the Dsl1 complex, Tip20p, may also contribute to COPI binding (Diefenbacher et al., 2011). Curiously, all homologs of Dsl1p from aquatic fungi, from plants and metazoans are lacking the lasso domain (Hirose et al., 2004; Schmitt, 2010). Instead, in mammalian cells an additional protein called UVRAG is required for the interaction of the COPI coat with the NRZ complex (He et al., 2013). UVRAG was hitherto known for its role in endosomal transport and autophagy as part of the Vps34 phosphatidylinositol 3-kinase complex II. According to the new data, it is able to bind phosphoinositides, and it can bind to RINT-1, the mammalian homolog of Tip20p, to perform an additional role in Golgi-ER transport (He et al., 2013). The UVRAG homolog in yeast, Vps38p, is involved vacuolar protein sorting, and it has not been found to act at the ER or to be involved in autophagy (Kihara et al., 2001). Thus, the mechanism of COPI/ER tether interaction differs between yeast and mammalian cells, but it is found, in variation, across species boundaries.

The binding of AP-3 vesicles to the Vps41p **HOPS** subunit was discovered by Rehling et al. (1999). Angers and Merz later showed that the whole HOPS tethering complex, and not just the Vps41p subunit alone, is involved in the interaction (Angers and Merz, 2009). In metazoans, the phenotypes of AP-3 and HOPS mutants are quite similar, indicating that the proteins act in a similar pathway (Zlatic et al., 2011a). There are, however, several discrepancies: (i) Unlike those from mammalian cells, AP-3 vesicles in yeast do not carry an outer cage layer consisting of clathrin. (ii) The units from mammalian HOPS are found to associate with AP-3 adaptor proteins as well as clathrin subunits (Zlatic et al., 2011b). (iii) AP-3 vesicles in yeast and mammalian cells are destined for the lysosome. They form, however, at different organelles, the late Golgi in yeast and a tubular early endosome in mammalian cells (Dell'Angelica, 2009). (iv) In mammalian cells, the HOPS complex is recruited to sites of AP-3 vesicle formation, while in yeast it is recruited to the target membrane of the AP-3 vesicles, the lysosome as well as the late endosomes. (v) Most relevant for the context of this review is that Vps41 from metazoans lacks the motif for AP-3 binding and the ALPS domain for lipid binding (Cabrera et al., 2010). However, a direct TGN to late endosome transport route for lysosomal proteins was recently described that does not require clathrin or the AP-1 adaptor complex. The vesicles that mediate this transport carry hVps41 and the SNARE VAMP7 (Pols et al., 2013). This shows a considerable diversification in HOPS complex recruitment, but nevertheless its tethering function appears to have remained conserved.

## Tethers and coats share structural motifs

**Figure 3:**
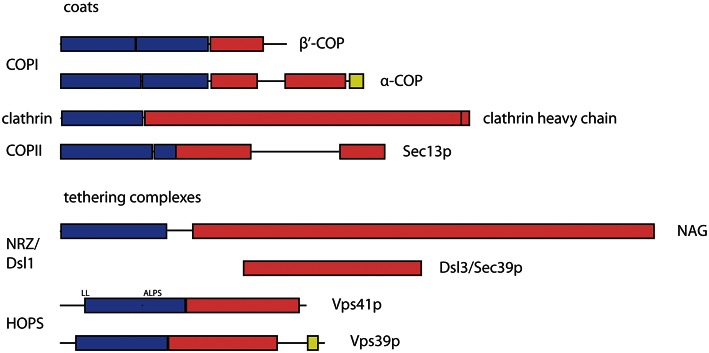
**Predicted and observed arrangement of structural protocoatomer elements in subunits of coat and tethering complexes**. The protocoatomer was identified as a potentially membrane-curving protein module in proteins of the nuclear pore complex and coat complexes (Devos et al., 2006). Blue color indicates β-propellers or WD40 repeats, while α-solenoids are indicated by red color. RING like domains (yellow) were found near the C-termini of α-COP and several subunits of the HOPS and CORVET complexes (Vps39p as shown above and in Vps8p, Vps11p and Vps18p; Nickerson et al., 2009; Kaur and Subramanian, 2015). According to the protocoatomer theory, the β-α arrangement indicates a common evolutionary origin of coat complexes. Its occurrence in tethering complexes may indicate that they also share a common origin with subunits of the coat and nuclear pore complexes. The domain organization of coat complexes was deduced from the structural data (Fath et al., 2007; Lee and Goldberg, 2010). For NAG, the boundaries of the domains were depicted as determined by Civril et al. (2010). The Dsl3/Sec39 protein is positioned below its mammalian homolog NAG (neuroblastoma amplified gene) in a region where both share some sequence similarities. The diagrams illustrating the domain organization and specific binding sites of the HOPS subunits were depicted as proposed by Nickerson et al. (2009). ALPS, amphiphilic lipid-packing sensor; RING, Really Interesting New Gene; WD40, 40 residue long repeat that ends with a tryptophan—aspartic acid motif; NAG, neuroblastoma amplified gene.

The last paragraphs have focused on functional interactions between coats and tethers, and have found an, if not ubiquitous, but notable range of interactions. Intriguingly, not only the interactions found in several transport routes speak for an interconnection between coats and tethers. Additionally, recently solved protein structures have revealed a surprising correspondence in structural motifs between coats and tethers. The protocoatomer architecture, an N-terminal 7-bladed β-propeller followed by extended α-solenoids or α-zigzag linker, is found in many subunits of coat complexes (i.e., clathrin, Sec13p, Sec31p, α-COP and β′-COP) as well as in nuclear porins (ter Haar et al., 1998; Fath et al., 2007; Lee and Goldberg, 2010). This β-α-fold architecture seems to be common to proteins that can bend membranes (Devos et al., 2006). Remarkably, the β-α-fold motif was also predicted for subunits of some multisubunit tethering complexes, for most of the HOPS subunits (including Vps3p and Vps8p from the related CORVET complex) and the NAG subunit of the mammalian Dsl1/NRZ complex (Nickerson et al., 2009; Civril et al., 2010; Figure 3). Experimental proof for its occurrence in tethers was obtained for the Vps18 subunit of the HOPS complex (Behrmann et al., 2014).

The β-propellers of clathrin heavy chains and of COPI subunits are oriented toward the membrane (Kirchhausen and Harrison, 1984; Dodonova et al., 2015). Similarly, the β-propellers of the HOPS subunits Vps41p and Vps18p were shown to bind to lipids (Cabrera et al., 2010; Behrmann et al., 2014). In the Dsl1 complex of yeast the Dsl3/Sec39 protein, a long a-solenoid, is also oriented with its N-terminus towards the membrane (Ren et al., 2009). This would also position the β-propeller present in the mammalian Dsl3/Sec39 homolog NAG (neuroblastoma amplified gene) close to the membrane (Civril et al., 2010). Since NAG shares all structural elements with another protein, ROD, that acts together with ZW10 and a third component in the recruitment of microtubules to the kinetochore, the β-α architecture must be an ancient property of the Dsl3/NAG proteins (Civril et al., 2010; Schmitt, 2010). Fungi very likely lost the β-propeller encoding region from the corresponding gene (Schmitt, 2010).

Recently, an additional similarity between COPI and HOPS/CORVET subunits was noted. At the C-termini of Vps11p, Vps18p, Vps8p,Vps39p, mammalian Vps41p and α-COP from many different species, Zn^2+^-binding RING domains were detected (Nickerson et al., 2009; Balderhaar and Ungermann, 2013; Kaur and Subramanian, 2015). For Vps11p, Vps18p, Vps8p, and α-COP the importance of this domain has been proven (Eugster et al., 2000; Nickerson et al., 2009; Zink et al., 2009).

These common features between coat proteins and tethers make the hypothesis conceivable that the coats and tethers were more alike in an ancient eukaryote, where both formed a proteinaceous layer at the surface of intracellular membranes. From that, they diversified and developed either to the cage-forming proteins around the vesicle, or a layer of vesicle-capturing proteins at the target membrane.

## TRAPP I/COPII interactions

One of the best characterized coat/tether interactions is that between the TRAPP I complex and COPII coat (Yu et al., 2006; Cai et al., 2007). The ability to bind vesicle coats and its apparent steady-state Golgi localization suggested that the TRAPP I complex acts as a COPII vesicle tether (Kim et al., 2006). Recently, evidence has accumulated that the complex acts upstream of tethering.

In general, the TRAPP 1 complex shows a quite distinct behavior and structural features compared to the Dsl1 and HOPS complexes: (i) TRAPP I is recruited to free COPII vesicles (Cai et al., 2007). It appears Golgi-localized at steady state since COPII vesicles fuse with their target membrane very quickly (Wang J. et al., 2015). (ii) There is no direct evidence for an interaction between the TRAPP I complex and SNAREs, even though many genetic interactions of a *bet3* mutation with SNARE-encoding genes have been described (Sacher et al., 2000). (iii) The TRAPP I complex is an activator (GEF) of the ER-Golgi-specific Rab/Ypt-GTPase Ypt1p (Jones et al., 2000; Wang et al., 2000), rather than a GTPase effector like for instance Vps41p of the HOPS complex. Accordingly, the TRAPP I complex does not need Ypt1p for its recruitment to vesicles.

The following observations indicate that the TRAPP I complex acts before the actual tethering step. The TRAPP I subunit Bet3p binds to the same site at the COPII subunit Sec23p that is also the binding site for Sar1p, the GTPase that triggers coat formation (Cai et al., 2007; Lord et al., 2011). Notably, Bet3p and Sar1p are later displaced by the casein kinase Hrr25p (Lord et al., 2011). This kinase is activated by Ypt1p on the vesicles (Wang J. et al., 2015), and phosphorylation of Sec23p and Sec24p by Hrr25p is required though not sufficient for uncoating (Lord et al., 2011). The phosphorylation state of Sec23p determines whether the coat subunit is ready for vesicle fusion or vesicle formation (Murakami et al., 1999; Dudognon et al., 2004; Lord et al., 2011; Bhandari et al., 2013). Taken together, these findings suggests that the TRAPP I binding represents an intermediate step of vesicle maturation that occurs well before the actual tethering step, and that the complex is released before COPII vesicles reach the Golgi membrane. The function of the TRAPP 1 complex rather appears to lie in determining the directionality of transport. A more likely tether for COPII vesicles is the coiled-coil homodimer tether Uso1p/p115 (Waters et al., 1992; Cao et al., 1998; Allan et al., 2000). This tether is also activated by Ypt1p/Rab1 (Allan et al., 2000), and binds to the SNAREs rbet1 and sec22b (Wang T. et al., 2015). Since Bet1p and Sec22p are present on opposing membranes in yeast (Parlati et al., 2000), Uso1p/p115 may bridge the gap between the membranes by interacting with single SNAREs at the vesicle and the target membrane (Grabski et al., 2012).

## Conclusions

To summarize, Dsl1/NRZ and HOPS complexes are representatives of tethering complexes that interact with coats. Both contain protocoatomer-like subunits. This indicates that these two tethers could be derived from primordial coat complexes. In ancestral eukaryotic cells, both donor and acceptor membranes may have been covered by different coats and fusion may have been initiated by the direct contact between them. During evolution, one of these coats acquired and improved its capability to induce membrane curvature, while the other with preference for flat membranes developed into a tethering factor. In line with this theory, the HOPS complex can bind to flat and curved membranes in a regulated manner, but it cannot induce curvature (Cabrera et al., 2010). The Dsl1/NRZ complex, in contrast, is a mixture of protocoatomer and CATCHR subunits. Notably, those two tethering complexes that share some coat characteristics are able to bind coats. Since these interactions were not conserved in metazoans or require additional binding partners, it is not clear whether the coat/tether interactions described here represent remainders of an ancient fusion mechanism, or whether fungi simply reinvented this tethering mode. The fact that they are still in operation and can be used for specific targeting purposes indicates that coat/coat contacts could indeed be considered as a part of an ancient fusion mechanism. This notion is in line with the organellar paralogy model (Dacks and Field, 2007). Coat/tether interaction can be useful in preventing premature mixing of different transport routes (Cabrera et al., 2010) or to keep membrane domains, where COPI vesicles arrive, separated from those where COPII vesicles form (Zink et al., 2009). Of course, more research has to be done to find whether other coat/tether interactions have specialized functions. At least one example exists where the p115 tether is involved in the regulation of COPI coat formation at the Golgi (Guo and Linstedt, 2013). The structure determination of other tethering factors will be extremely helpful in determining whether they too share motifs with coats.

## Author contributions

All authors listed, have made substantial, direct and intellectual contribution to the work, and approved it for publication.

## Funding

SB was supported by a stipend from the Max Planck Society and SS received an “excellent stipend” from the GGNB graduate school.

### Conflict of interest statement

The authors declare that the research was conducted in the absence of any commercial or financial relationships that could be construed as a potential conflict of interest.
