# Corrigendum: Coat/Tether Interactions-Exception or Rule?

**DOI:** 10.3389/fcell.2016.00090

**Published:** 2016-08-30

**Authors:** Saskia Schröter, Sabrina Beckmann, Hans Dieter Schmitt

**Affiliations:** Neurobiology, Max Planck Institute for Biophysical ChemistryGöttingen, Germany

**Keywords:** vesicle trafficking, coat complexes, protocoatomer, coated vesicles, tethering complex

We would like to correct the spelling of the last name of the author Saskia Schröter. It was misspelled as “Schroeter,” whereas the correct spelling is “Schröter.” This mistake does not affect the scientific conclusions of the article in any way. The authors apologize for this oversight.

The original article has been updated.

## Author contributions

All authors listed, have made substantial, direct and intellectual contribution to the work, and approved it for publication.

### Conflict of interest statement

The authors declare that the research was conducted in the absence of any commercial or financial relationships that could be construed as a potential conflict of interest.

